# SARS CoV-2-Induced Viral Sepsis: The Role of Gut Barrier Dysfunction

**DOI:** 10.3390/microorganisms10051050

**Published:** 2022-05-19

**Authors:** Stelios F. Assimakopoulos, Gerasimos Eleftheriotis, Maria Lagadinou, Vassilios Karamouzos, Periklis Dousdampanis, Georgios Siakallis, Markos Marangos

**Affiliations:** 1 Division of Infectious Diseases, Department of Internal Medicine, University of Patras Medical School, 26504 Patras, Greece; makiseleftheriotis@yahoo.gr (G.E.); mlagad@upatras.gr (M.L.); vkaramouzos@hotmail.com (V.K.); marangos@upatras.gr (M.M.); 2 Hemodialysis Unit Kyanos Stavros, 26225 Patras, Greece; dousdampanis@yahoo.gr; 3 Department of Basic and Clinical Sciences, University of Nicosia Medical School, Nicosia 2408, Cyprus; siakallis.g@med.unic.ac.cy

**Keywords:** SARS-CoV-2, COVID-19, intestinal barrier, microbiota, tight junctions, microbial translocation, bacterial translocation, endotoxin, sepsis, viral sepsis

## Abstract

A considerable proportion of patients with severe COVID-19 meet Sepsis-3 criteria and share common pathophysiological mechanisms of multiorgan injury with bacterial sepsis, in absence of secondary bacterial infections, a process characterized as “viral sepsis”. The intestinal barrier exerts a central role in the pathophysiological sequence of events that lead from SARS-CoV-2 infection to severe systemic complications. Accumulating evidence suggests that SARS-CoV-2 disrupts the integrity of the biological, mechanical and immunological gut barrier. Specifically, microbiota diversity and beneficial bacteria population are reduced, concurrently with overgrowth of pathogenic bacteria (dysbiosis). Enterocytes’ tight junctions (TJs) are disrupted, and the apoptotic death of intestinal epithelial cells is increased leading to increased gut permeability. In addition, mucosal CD4(+) and CD8(+) T cells, Th17 cells, neutrophils, dendritic cells and macrophages are activated, and T-regulatory cells are decreased, thus promoting an overactivated immune response, which further injures the intestinal epithelium. This dysfunctional gut barrier in SARS-CoV-2 infection permits the escape of luminal bacteria, fungi and endotoxin to normally sterile extraintestinal sites and the systemic circulation. Pre-existing gut barrier dysfunction and endotoxemia in patients with comorbidities including cardiovascular disease, obesity, diabetes and immunosuppression predisposes to aggravated endotoxemia. Bacterial and endotoxin translocation promote the systemic inflammation and immune activation, which characterize the SARS-CoV-2 induced “viral sepsis” syndrome associated with multisystemic complications of severe COVID-19.

## 1. Introduction

Although the clinical course of COVID-19 is usually mild, a small proportion of patients might develop severe disease associated with acute respiratory distress syndrome (ARDS), multiple organ failure and increased mortality. In this severe form of COVID-19 there is excessive release of proinflammatory cytokines such as interleukin (IL)-1β, IL-2, IL-6, IL-7, tumor necrosis factor (TNF)-α, interferon-γ inducible protein 10, granulocyte colony stimulating factor, monocyte chemoattractant protein 1 and macrophage inflammatory protein 1-α, characterized as a “cytokine storm” syndrome [1]. These inflammatory mediators promote multiple organ injury affecting mainly the lung, but also the heart, endothelium, brain, kidney, liver, pancreas and intestine. Sepsis is defined as life-threatening organ dysfunction caused by a dysregulated host response to infection [2]. According to the Sepsis-3 criteria, most of these seriously ill COVID-19 patients have sepsis fulfilling a Sequential Organ Failure Assessment (SOFA) score of 2 points or more [2]. Even though sepsis may occur as a host response regardless of the type of infectious agent, on clinical grounds sepsis is usually reported as a complication of bacterial infections. Furthermore, the sepsis syndrome associated with COVID-19 seems to be underestimated and underreported in several studies [3]. Nevertheless, bacterial coinfections or secondary infections are present in a low proportion of COVID-19 patients [4]. The higher incidence of bacterial infections after administration of dexamethasone or anti-cytokine therapies does not provide supportive evidence of the bacterial etiology of sepsis in COVID-19 patients, as the SARS-CoV2-associated sepsis is usually already evident before institution of these immunosuppressive treatments [5]. Therefore, an increasing amount of evidence, demonstrates that a significant proportion of COVID-19 patients meet Sepsis-3 criteria and the underlying pathophysiological mechanisms of multiorgan injury, such as dysregulated immune system, endothelial cell damage, thromboinflammation and tissue damage by neutrophils, monocytes and lymphocyte may be, at least partly, common with bacterial sepsis in a process characterized as “viral sepsis” [3].

## 2. The Gut Barrier in Sepsis

The gut barrier function is comprised by three major levels of defence [6]: (A) The biological barrier, (B) the mechanical barrier and (C) the immune barrier. The biological barrier is made up of resident intestinal flora (gut microbiota). Normal gut flora exerts important metabolic, immunological and gut-protective functions. Microbiota exert metabolic actions fermenting carbohydrates and indigestible oligosaccharides, complete the entero-hepatic cycle of biliary acids and synthesize vitamins B, K and short chain fatty acids (SCFA) which provide energy to the intestinal epithelium [7]. Immunologically, the gut microbiota is a continuous crosstalk with both the innate and adaptive intestinal immune systems through production of pathogen-associated molecular patterns (PAMPs), which interact with intestinal immune cells through specific receptors, thus contributing to gut immunomodulation [8]. In addition, the intestinal microbiota resists colonization by potentially pathogenic bacteria and prevents their growth through antagonism for nutrients. The mechanical barrier is consisted by intestinal epithelial cells and their specific structures named “tight junctions” (TJs), which tightly interconnect them in the most apical part of the lateral cell membranes. TJs control the passage of ions, molecules and cells through the paracellular space [6,9]. Capillary endothelial cells, which are also interconnected by TJs, form the second line of the mechanical barrier defense to pathogen invasion. The immune barrier is composed of gut associated lymphoid tissue (GALT), IgA producing B (plasma) cells, effector and T-regulatory (Treg) cells, Group 3 innate lymphoid cells, resident macrophages and dendritic cells in the lamina propria. The continuous interplay of gut microbiota with the intestinal immune system drives the development of tolerance to commensal bacteria and effective immunological response to potential microbial invaders [10]. Exposure of immune cells to microbial pathogens and/or their products leads to B cell switch to IgA class, Tregs induction and T cell differentiation to Th17 [8].

The intestinal barrier is characterized as the “motor” of sepsis, exerting a central pathophysiological role in the development of multiple organ dysfunction [11]. Numerous experimental and clinical studies have demonstrated that all the three levels of gut barrier defense are compromised in sepsis [11,12,13]. Specifically, the intestinal microflora equilibrium is disrupted, there is increased apoptosis of intestinal epithelial cells and reduced expression of TJs, Paneth cells and submucosal Treg cells are decreased while intraepithelial CD3(+) T-lymphocytes and proinflammatory cytokines are increased. These gut barrier alterations promote bacterial and endotoxin translocation in the systemic circulation activating the release of proinflammatory mediators, which may cause deleterious effects on the structure and function of remote organs, resulting in multiple organ dysfunction [13].

## 3. SARS-CoV-2 Infection and the Intestinal Barrier

**Figure 1:**
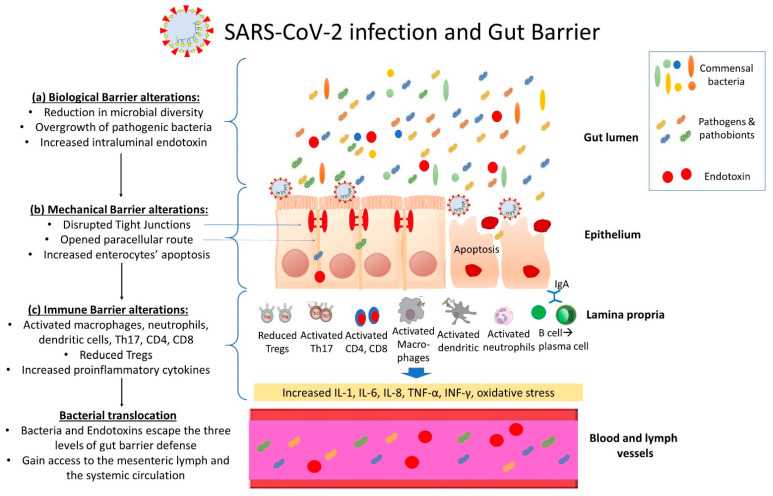
**Overview of intestinal barrier dysfunction in SARS-CoV-2 infection:** SARS-CoV-2 infection induces a multifactorial disruption of intestinal barrier integrity by negatively affecting all of its major levels of defense; (a) The biological barrier, consisting of the commensal gut microbiota, is disrupted with reduction in microbial diversity and beneficial bacteria population such as butyrate-producing firmicutes concurrently with overgrowth of pathogenic bacteria (dysbiosis), while luminal endotoxin is increased. Intestinal dysbiosis through a continuous crosstalk with the intestinal epithelium negatively affect the integrity of the mechanical and immune barriers. (b) The mechanical barrier, which is comprised of the intestinal epithelial cells and their close interconnections (tight junctions—TJs), is also compromised. SARS-CoV-2 disrupts enterocytes’ TJs thus opening the paracellular route and additionally promotes intestinal epithelial cells apoptosis. (c) The intestinal immune barrier is characterized by activation of mucosal CD4(+) and CD8(+) T cells, Th17 cells, decreased T-regulatory cells and activated neutrophils, dendritic cells and macrophages, leading to overproduction of proinflammatory cytokines and reactive oxygen species, associated with tissue injury and further disruption of the integrity of the mechanical barrier. Through this dysfunctional intestinal barrier, microbes and endotoxin can escape the intestinal lumen and gain access to the systemic circulation (bacterial and endotoxin translocation).

There is increasing evidence that SARS-CoV-2 infection disrupts the integrity of the intestinal barrier function, by negatively affecting all levels of defense; the biological, mechanical and immunological barrier (Figure 1):

### 3.1. SARS-CoV-2 and the Intestinal Biological Barrier

The intestine is the largest immune organ of the human body. It contains approximately 10^14^ resident bacteria, collectively known as gut microbiota. Gut microbiota are an important factor in the regulation of immune response against infectious agents. Many studies in patients with COVID-19, using either shotgun metagenomic sequencing or 16S rRNA sequencing, have demonstrated significant alterations in gut microbiome composition. These alterations were irrespective of antibiotic administration, which is another major risk factor for gut dysbiosis [14,15,16,17]. Furthermore, gut microbiota composition alterations remain even after recovery from COVID-19 [14].

At the phylum level, several studies have shown that *Bacteroidetes* are more abundant in COVID-19 patients than in healthy controls [14,15]. Moreover, relative abundance of *Firmicutes* and *Actinobacteria* is subnormal, even though *Firmicutes* remains the most abundant phylum [14,15]. In other words, these patients have a low *Firmicutes/Bacteroidetes* ratio, which is indicative of dysbiosis. At the species level, enrichment of *Bacteroides dorei* and *Firmicutes* such as *Ruminococcus gnavus* and *Ruminococcus torques* has been observed. On the other hand, there is depletion of *Bifidobacterium adolescentis*, which belongs to *Actinobacteria*, and short-chain fatty acid (SCFA)-producing *Firmicutes* such as *Faecalibacterium prausnitzii* and *Eubacterium rectale* [15,16,18,19]. *E. rectale*, *Bifidobacteria* and *F. prausnitzii* were also underrepresented in samples collected after disease resolution [15,20]. Furthermore, patients with COVID-19 presented microbiota enrichment with opportunistic pathogens such as *Clostridium hathewayi*, *Clostridium ramosum*, *Actinomyces viscosus*, *Streptococcus* and *Rothia* compared to healthy individuals; more virulent bacteria such as *Enterococci* were also enriched [19,21,22].

*Ruminococcus gnavus* and *Ruminococcus torques* are bacteria that can use mucins as energy source and for that reason they are also increased in inflammatory bowel disease [23]. On the other hand, SCFA-producing bacteria are known for their potential in mitigating the inflammatory immune response as they can reduce interleukin (IL)-8, IL-12 and interferon (IFN)-γ production and block nuclear factor-κB activation [24]. An in vitro study has demonstrated that SCFAs promote differentiation of T cells into both Tregs and T helper (Th17 and Th1) cells, depending on the circumstances [25]. Presence of SCFAs in high abundance has been related with low infectivity of fecal samples from SARS-CoV-2-positive patients [26]. *F. prausnitzii* in particular, which is the most abundant bacterium in the gut microbiota of healthy adults, promotes the secretion of the anti-inflammatory IL-10 by inducing Tregs in the colonic mucosa of patients with Crohn’s disease and animal models of colitis [27,28]. Therefore, it is not surprising that an inverse correlation has been found between abundance of *F. prausnitzii* and COVID-19 severity [21]. *Bifidobacteria* are not able to produce SCFA per se; however, they contribute to SCFA production through cross-feed of SCFA-producing bacteria with their end-products such as acetate and lactate [29].

Notably, it seems that patients with critical illness due to SARS-CoV-2 present a distinct pattern of gut microbiota. Gaibani et al., compared gut microbiota from critically ill patients due to COVID-19 or other etiologies and found that *Enterococcus* spp. were present almost exclusively in COVID-19 patients, especially in those who developed bacteremia [22]. Almost half of these enterococcal infections were caused by *E. faecium* [22]. This finding was in alignment with the results of observational studies from Italy reporting high prevalence of enterococcal bloodstream infections in critically ill COVID-19 patients [30,31]. The altered gut microbiota of SARS-CoV-2-positive individuals may be also a risk factor for pneumonia or other superinfections, as observed in other viral infections such as avian H7N9 influenza [32]. Concerning bacterial richness and diversity during acute SARS-CoV-2 infection, the use of Chao1 and Shannon index, respectively, provided conflicting results [14,16,17,19,22,33,34,35]. Regarding alpha diversity, this marker was associated with disease severity because a significant reduction was found in COVID-19 patients with hypoxemia compared to non-hypoxemic patients [33]. Reduction in bacterial richness was more profound in seriously ill patients who were admitted in the intensive care unit [36]. In addition, low Shannon diversity index seems to be a risk factor for high-flow oxygen requirement or mechanical ventilation (noninvasive or invasive) during hospitalization for COVID-19 [37].

The distinct gut microbiota composition can remain for over six months after infection. This persistent gut dysbiosis has been associated with long COVID [16,38]. Persistent respiratory symptoms were positively correlated with *Streptococci*; *Faecalibacterium prausnitzii* was negatively correlated with chest tightness after activity, even though all patients had pulmonary function tests within normal limits [16,38]. Liu et al., investigated bacterial diversity and richness six months after hospitalization for COVID-19 and found that these factors were significantly lower in patients with persistent long COVID symptoms as compared to asymptomatic patients or controls [16].

### 3.2. SARS-CoV-2 and the Intestinal Mechanical Barrier

Intestinal epithelial cells express the angiotensin-converting enzyme 2 receptor and can be infected by the SARS-CoV-2 virus (4). Previous studies have shown that SARS-CoV-2 after its entry to intestinal epithelial cells is able to replicate [39]. SARS-CoV-2 RNA is commonly detected in feces from COVID-19 patients, while risk of detection is threefold in those with gastrointestinal dysfunction [40]. Interaction of SARS-CoV-2 with ACE2 in intestinal epithelial cells may alter the expression and function of the TJ proteins thus leading to disruption of the paracellular barrier function [41,42]. In animal models, altered expression of ACE2 has been associated with disrupted gut barrier function [43].

In a recently published study, we have shown that patients with COVID-19-associated pneumonia have significantly higher serum ZO-1 concentrations as compared to healthy controls and this is associated with endotoxin translocation [44]. ZO-1 is a 210–225 kDa phosphoprotein that interacts with (i) the TJ proteins occludin, claudins and junctional adhesion molecule, (ii) molecular components of the intracellular tight junctional plaque such as ZO-2, ZO-3, afadin-6, cingulin and (iii) the actin cytoskeleton [9]. Therefore, it plays a key role in the structural and functional integrity of the paracellular barrier by bringing and connecting TJ proteins to the cytoskeleton [9]. ZO-1 levels in serum are inversely related to its intestinal expression and serum ZO-1 is an established marker of increased intestinal paracellular permeability in diverse pathological conditions such as in inflammatory bowel disease, celiac disease, diabetes type 1, obesity and rheumatoid arthritis [45,46,47]. In another very interesting study with application of multi-omic system biology approach, it was shown that severe COVID-19 is associated with increased blood concentrations of the TJ permeability markers ZO-1 and occluding, concurrently with detection of translocated bacterial and fungal products in the systemic circulation. In addition, a SARS-CoV-2 infection model on a chip for the study of the intestinal pathophysiology, demonstrated the disruption of the intestinal barrier integrity evidenced by morphological injury of intestinal villi, dispersed distribution of mucus-secreting cells and reduced expression of adhesion molecules (E-cadherin) [48]. Moreover, the vascular endothelium exhibited abnormal cell morphology, with disrupted adherent junctions which indicates disruption of the endothelial barrier as well.

Another important factor of the intestinal mechanical barrier integrity is enterocytes’ apoptosis. A previous immunohistochemical study in COVID-19 patients detected significantly increased numbers of cleaved caspase-3(+) apoptotic epithelial cells [49]. Highly proliferating epithelial cells were also seen indicating a regenerative response to intestinal injury. SARS-CoV-2 infection of enterocytes induces the expression of proinflammatory cytokines similar to that observed in lung epithelial cells [50], and activates the intraepithelial CD8^+^ T cells [49]. These immunological alterations might negatively affect the mechanical barrier integrity through promotion of the intestinal epithelial cell apoptosis and/or through downregulation of enterocytes’ TJs [6]. Alternatively, lung infection and injury by SARS-CoV-2 leads to the systemic release of proinflammatory cytokines which disrupt the integrity of the gut barrier.

### 3.3. SARS-CoV-2 and the Intestinal Immune Barrier

SARS-CoV-2 infection in animal models and humans has been associated with induction of a proinflammatory milieu consisted by high levels of IL-1, IL-6, IL-8 and IFN-γ and low levels of the anti-inflammatory IL-10, detected in the small and large intestinal tissue and feces [51,52]. IFN-γ is produced by multiple types of immune cells, particularly Th1 T cells, and is one of the main inducers of cellular immune response to infection, through activation of macrophages, enhanced antigen presentation and T cell differentiation [53]. IFN-γ can also interact with epithelial cells directly, leading to expression of chemokines and secretion of antimicrobial peptides such as defensins and lysozyme [54,55]. On the other hand, human intestinal epithelial cells after SARS-CoV-2 infection produce only type III IFN; pretreatment of colon organoids with IFN-β1 and IFN-λ led to significantly milder infection [50].

One of the main mechanisms for inhibition of viral entry in epithelial cells, including intestinal Paneth cells, is production of antimicrobial and immunomodulatory peptides such as defensins, which are also produced by neutrophils. Defensins have the ability to recruit and activate monocytes, naive T cells and immature dendritic cells [56,57]. In particular, it has been previously shown that defensin 5 interacts with ACE2 receptor and does not allow binding of SARS-CoV-2 [58]. These results were corroborated by another study, which revealed that pretreatment of cells with human defensin 5 had a moderate protective role against infection from the B.1.1.7 SARS-CoV-2 variant, whereas no positive effect was observed when defensin 5 was administered after infection [59]. Defensin 5, however, has to be already in adequate concentrations before SARS-CoV-2 lands to intestinal cells in order to exert its protective role, because the virus has higher affinity for ACE2 receptor [58].

The main evidence about changes in mucosal immune cells during infection by SARS-CoV-2 comes from studies that used gastrointestinal tract biopsies from patients who had been recently infected, including post-mortem samples. Wang et al. performed a cytometric tissue analysis of deceased individuals from COVID-19, and showed that monocytes, as well as CD11b+ macrophage, CD11c+ dendritic cell, natural killer cell and B cell counts were significantly higher in the intestine of these patients as compared to controls [60]. Monocytes/macrophages with high expression of CD68 and CD14, indicating their recent recruitment from the periphery, were detected in the duodenal mucosa of five SARS-CoV-2 infected patients who underwent an endoscopy without macroscopic abnormalities after an average time of 8.2 days following the onset of COVID-19 symptoms [49]. Other microscopic features included an increase in mucosal CD4+ T cells and accumulation of antigen-experienced, activated intraepithelial CD8+ T cells [49]. Increase in intraepithelial lymphocytes and in lamina propria T cells was also observed at 10/17 of gastrointestinal biopsies taken from patients after the first month of the last positive SARS-CoV-2 test [61]. In addition, effector (PD-1+, CD38+) CD4+ (which are playing a key role for mucosal immunity) and CD8+ T cells and CD8+CD103+ T cells (tissue resident memory) were increased in gastrointestinal biopsies of COVID-19 recovered individuals as compared to controls [61]. The Th17 cells, which are subpopulations of CD4+ T cells producing IL-17, stimulate enterocytes’ proliferation, antibacterial defensins expression and recruiting of neutrophils to the gut-associated lymphoid tissue to clear bacterial pathogens and endotoxin [62,63]. In SARS-CoV-2 infection, especially in severe cases, Th17 cells have been found overactivated [64]. On the other hand, previous studies have shown that mucosal Tregs are significantly decreased, especially in patients with severe disease, and this is associated with immune dysregulation [52]. The imbalance between Th17/Tregs response has been associated with tissue injurious overactive immune responses [64].

Another element of the intestinal immunological barrier is secretory IgA (sIgA), which is a possible explanation for the more limited inflammatory response that SARS-CoV-2 can initiate in the intestine compared to the lungs. sIgA is the main topically acting immunoglobulin in the intestine, as opposed to the lungs where IgG antibodies mainly act, and has the ability to bind and neutralize antigens without causing inflammation because of its inability to activate the complement cascade [65]. Furthermore, secretory, dimeric IgA is more potent in viral neutralization than IgG [66].

## 4. Gut Barrier Dysfunction, Systemic Inflammation and Immune Activation

Previous studies have shown that SARS-CoV-2 infection is associated with increased bacterial translocation due to intestinal barrier dysfunction. Specifically, it has been shown that COVID-19 patients present significantly increased levels of circulating lipopolysaccharide (LPS) and lipopolysaccharide binding protein (LBP) [44,67]. Moreover, in patients with severe COVID-19, there is evidence of circulating bacteriome as detected by 16s RNA gene amplification technique, attributed to bacterial translocation form the gut since secondary bacterial infections are rare [68]. Another study investigated the possible interconnection of the organisms identified in the blood of COVID-19 patients with their gut microbiome and demonstrated a close linking [69]. Patients with severe COVID-19 also presented high levels of β-glucan, a polysaccharide component of the cell wall of most fungal species, and a marker of fungal translocation [67].

**Figure 2:**
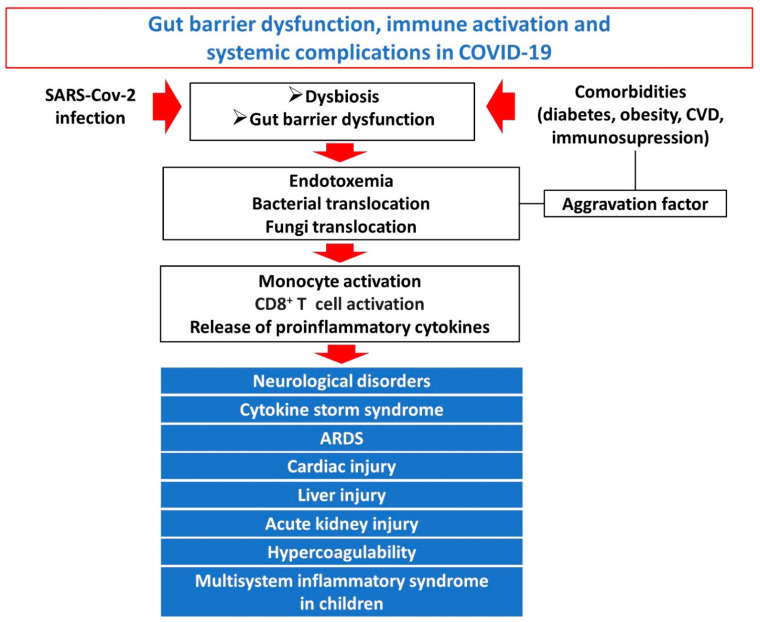
**Overview of the interconnection between gut barrier dysfunction, bacterial and endotoxin translocation, immune activation and systemic complications of severe COVID-19:** the dysfunctional gut barrier in SARS-CoV-2 infection permits the escape of luminal bacteria, fungi and endotoxin to normally sterile extraintestinal sites and the systemic circulation. Pre-existing gut barrier dysfunction and endotoxemia in patients with comorbidities such as cardiovascular disease, obesity, diabetes and immunosuppression predisposes to aggravated endotoxemia. Bacterial and endotoxin translocation induce monocyte and CD8+ T cell activation and release of proinflammatory cytokines. Systemic inflammation and immune activation characterize the SARS-CoV-2 induced “viral sepsis” syndrome, which is associated with multisystemic complications of severe COVID-19.

Bacterial and endotoxin translocation in COVID-19 induces a significant increase in several markers of systemic inflammation, such as IL-6, IL-1b, IL-8, MCP-1, IP-10 and TNF-a [67,70]. Moreover, the plasma levels of soluble CD14 (sCD14), a marker of monocyte activation, was significantly increased. Interestingly, in severe COVID-19 intestinal barrier dysfunction and bacterial translocation markers correlated strongly with markers of systemic inflammation and immune activation [67]. Specifically, an increase in HLA-DR+CD38+CD8+ T cells has been previously demonstrated combined with increase in the pro-inflammatory CCR4+CCR6+Th17 cells and decreased CD3+CD8+suppressor T cells and CD3+CD4+ helper-T cells [71]. According to a more detailed analysis of T cell subpopulations in severe COVID-19 patients the HLA-DR+CD38+CD8+ T population consisted of two diverse subsets with distinct characteristics: HLA-DR+CD38^dim^ and HLA-DR+CD38^hi^. The HLA-DR+CD38^hi^CD8+ T cells are overactivated and dysregulated, as evidenced by expression of multiple inhibitory and stimulatory checkpoints, higher apoptosis and impaired killing capacity, and associated with systemic inflammation, tissue injury and immune disorders of severe COVID-19 patients [70]. Systemic immune activation in COVID-19, driven at least partly by gut barrier dysfunction and bacterial translocation, has been associated with all major systemic complications of COVID-19 including the cytokine storm syndrome, acute respiratory distress syndrome, cardiac dysfunction, hypercoagulability, neurological dysfunction, kidney injury and the multisystem inflammatory syndrome in children [72]. Pre-existing gut barrier dysfunction and endotoxemia in patients with comorbidities such as cardiovascular disease, obesity, diabetes, immunosuppression predisposes to aggravated endotoxemia and endotoxin-induced immune activation after SARS-CoV-2 infection contributing to higher rates of severe COVID-19 in these patients [73]. A potential mechanism may be that SARS-CoV-2 infection induces ACE2 deficiency leading to induced endotoxemia and deteriorated endotoxin tolerance [73]. A schematic overview of the interconnection between gut barrier dysfunction, bacterial and endotoxin translocation, immune activation and systemic complications of severe COVID-19 is provided in Figure 2.

## 5. Therapeutic Approaches Targeting the Gut Barrier

Therapeutic approaches aiming at preventing or limiting the bacterial translocation process in COVID-19 patients can be divided into three major categories; (a) treatments aiming to preserve the biological barrier (normal intestinal microbiota) and/or inhibit pathogenic bacteria overgrowth and their increased attachment to the intestinal mucosa, which may initiate the bacterial translocation process, (b) therapies aiming at enhancing the integrity of the intestinal epithelial barrier by preventing gut mucosal injury and (c) therapies aiming at restoration of the intestinal immune barrier function.

### 5.1. Therapies Aiming at Restoration of the Intestinal Biological Barrier

Preservation of intestinal microbiota equilibrium with the use of **probiotics, prebiotics and synbiotics** is an important treatment option. Probiotics are living non-pathogenic microorganisms, which when administered in optimum amounts promote a healthy gut microbiome associated with well-documented beneficial health effects, prebiotics are specific plant fibers that promote the growth of beneficial bacteria and synbiotics are a combination of the two [74]. In a Cochrane metanalysis of 13 randomized controlled trials involving 3720 patients, probiotics were effective in reducing episodes of upper respiratory tract infection, mean duration of symptoms and use of antibiotics [75]. A previous study with 156 severe COVID-19 patients showed that the administration of a triple probiotic formula consisted of *Bifidobacterium longum*, *Lactobacillus bulgaricus* and *Streptococcus thermophilus* improved diarrhea symptom and reduced the inflammatory markers procalcitonin (PCT) and C-reactive protein (CRP) [76]. In another study, a four-strain probiotic composition was associated with a significant increase in complete viral and symptomatic remission by day 30 in COVID-19outpatients. Some other studies on probiotics supplementation in COVID-19 patients with diarrhea showed that disease duration and gastrointestinal symptoms, such as abdominal distension, nausea and vomiting, were significantly improved [77]. Moreover, supplementation with *Ligilactobacillus salivarius* MP101 in elderly patients who tested positive for SARS-CoV-2 significantly decreased the concentrations of fecal BAFF/TNFSF13B, APRIL/TNFSF13, chitinase 3-like 1, IL32, IL34, gp130/sIL-6Rb, sTNF-R1 and sTNF-R2, which is consistent with amelioration of intestinal inflammation [78]. A novel gut microbiota-derived synbiotic formula known as SIM01, when administered as an adjuvant therapy in 25 COVID-19 patients, enhanced antibody formation against SARS-CoV-2 and reduced the inflammatory response, as measured by plasma interleukin (IL)-6, IL-1RA, monocyte chemoattractant protein (MCP-1), macrophage colony-stimulating factor (M-CSF) and TNF-α [79]. The specific effect of probiotics on gut barrier parameters and immune activation has not been elucidated up to now, but several different probiotic strains and combinations are currently tested in COVID-19 while more information is pending [80].

### 5.2. Therapies Aiming at Restoration of the Intestinal Mechanical Barrier

**“Immunonutrition”** is the enteral or parenteral administration of pharmacologically active nutrients that may beneficially modulate the inflammatory and metabolic response to diverse pathological insults such as surgery or critical illness and enhance immune function. The enteral administration of the basic nutrition elements enriched with immunomodulating substrates, is referred to as enteral immunonutrition, and has been shown to prevent gut barrier injury by supplying enterocytes with energy. The most well-studied immunonutrients are glutamine, arginine, ω-3 fatty acids, γ-linoleic acid and nucleotides [81]. In a recent study, 30 patients with COVID-19 were supplemented with oral L-Glutamine and compared with 30 COVID-19 patients without L-Glutamine supplementation. Enteral L-glutamine led to a shortened hospital stay and less ICU admissions [82]. In another study, addition of oral L-arginine to standard therapy in severe COVID-19 patients significantly decreased the length of hospitalization and reduced the respiratory support at 10 days after starting the treatment [83]. Supplementation with omega-3 fatty acids improved 30-day survival and several parameters of respiratory and renal function in critically ill patients with COVID-19 [84]. Immunonutrition has been shown to exert pleiotropic actions on the intestinal mucosa, including proliferative, antiapoptotic, antioxidant and anti-inflammatory effects, thus enhancing the mechanical (enterocytes and tight junctions) and immunological integrity of the gut barrier and preventing bacterial translocation [85,86,87]. In addition, previous studies have shown that the altered microbiota composition in COVID-19 is a source of high oxidative stress which may injure the intestinal epithelium [88]. Therefore, **antioxidant** treatments including α-tocopherol, ascorbic acid, allopurinol and N-acetyl-cysteine might have a positive impact on the mechanical barrier integrity through prevention of oxidative stress-induced apoptosis of enterocytes or TJs’ disruption [89].

### 5.3. Therapies Aiming at Restoration of the Intestinal Immune Barrier

Disruption of T-cell homeostasis is an important parameter of gut immune barrier dysfunction in COVID-19 and other viral infections including HIV infection. IL-7 promotes T cell development and homeostasis and previous studies have shown that administration of **recombinant IL-7** induced CD4+ T-cell recovery in HIV infected patients [90].

Another important feature of intestinal immune barrier alterations in COVID-19 is activation of lamina propria macrophages, which secrete proinflammatory mediators that promote enterocytes’ apoptosis and disruption of TJs leading to enhanced bacterial translocation and development of a local vicious cycle of inflammation [52]. In this perspective, the use of **glucocorticoids**, which exert potent anti-inflammatory actions, has a theoretical basis. Glucocorticoids use in diverse preclinical models of intestinal barrier injury has given contradictory results, and negative effects on gut barrier function have been attributed, at least partly, on their negative impact on mucus production and epithelial healing [91]. However, preclinical studies on sepsis have demonstrated a positive impact of glucocorticoids based on their anti-inflammatory action, and their TJs preserving effect [12]. In addition, dietary bioactive **phenolic compounds** (resveratrol, curcumin, quercetin) exert potent anti-inflammatory and antioxidant actions through NF-κB inhibition, which inhibits proinflammatory cytokines production [92]. Their protective action on gut barrier function has been previously shown in intestinal epithelial cell culture experiments and in animal models, while clinical data in COVID-19 patients are limited [92].

## 6. Conclusions

Patients with severe COVID-19 suffer from a “viral sepsis” syndrome and are prone to multiorgan dysfunction. According to recent pathophysiological theories, supported by preclinical and clinical evidence, the intestinal barrier exerts a central role in the sequence of events that lead from SARS-CoV-2 infection to the development of viral sepsis with severe systemic complications. SARS-CoV-2 disrupts the integrity of the biological, mechanical and immunological gut barrier with mechanisms described in the present review. This dysfunctional gut barrier permits the escape of luminal bacteria, fungi and endotoxin to normally sterile extraintestinal sites and the systemic circulation. Pre-existing gut barrier dysfunction and endotoxemia in patients with comorbidities including cardiovascular disease, obesity, diabetes and immunosuppression predisposes to aggravated endotoxemia. Bacterial and endotoxin translocation promote the systemic inflammation and immune activation, which characterize the SARS-CoV-2 induced “viral sepsis” syndrome associated with multisystemic complications of severe COVID-19. When treating patients with COVID-19-associated sepsis, we should not neglect protecting their intestinal barrier by applying general measures including adequate fluid replacement to prevent visceral-microcirculatory disturbances or enteral nutrition to provide important nutrients for enterocytes. Further clinical studies are needed to explore the potential positive impact of more specific gut barrier modulating treatments.
